# COVID-19 vaccine hesitancy and associated factors among infertile couples undergoing assisted reproductive treatment

**DOI:** 10.3389/fimmu.2022.973600

**Published:** 2022-09-27

**Authors:** Xinyan Wang, Huiyan Wang, Aijun Du, Junchao Wang, Jie Shi, Yunshan Zhang, Yinfeng Zhang, Junfang Ma, Wenjia Meng, Jiabei Lv, Haining Luo

**Affiliations:** Center for Reproductive Medicine, Tianjin Central Hospital of Obstetrics and Gynecology, Maternal Hospital of Nankai University, Tianjin Key Laboratory of Human Development and Reproductive Regulation, Tianjin, China

**Keywords:** COVID-19, vaccine hesitancy, infertility, assisted reproductive technology, China, primary vaccination, booster vaccination

## Abstract

Although periconception vaccination is important to maternal and neonatal health, little is known about the COVID-19 vaccine hesitancy among infertile couples seeking fertility treatment. Thus, we conducted this survey among infertile patients in a reproductive medicine center, between September 2021 and December 2021, to estimate the prevalence of COVID-19 vaccine hesitancy and its influencing factors. Information was collected through face-to-face interviews among volunteers. Among the 987 included interviewees, 17.33% reported hesitancy in primary vaccination, 25.63% reported hesitancy in booster vaccination, and 32.32% delayed the primary vaccination. Hesitancy in primary vaccination was associated with unexplained infertility (OR: 1.77, 95% CI: 1.05-2.98), ongoing IVF treatment (OR: 2.17, 95% CI: 1.22-3.89), concerns for vaccine safety (OR: 4.13, 95% CI: 2.66-6.42), effectiveness (OR: 1.62, 95% CI: 1.15-2.28), and influence on pregnancy (OR: 2.80, 95% CI: 1.68-4.67). These factors were also associated with hesitancy in booster vaccination. Delay of the primary vaccination was inversely associated with a college or above degree (OR: 0.49, 95% CI: 0.27-0.87), previous history of influenza vaccination (OR: 0.67, 95% CI: 0.46-0.98), and was positively associated with concerns for the influence on pregnancy (OR: 7.78, 95% CI: 5.01-12.07). It is necessary to carry out targeted education program by health professionals to publicize the benefits of periconception vaccination, and to reduce the resistance to COVID-19 vaccine among infertile couples.

## Introduction

Since the first report of the novel coronavirus disease-19 (COVID-19) in December 2019, the outbreak of this infectious disease caused by the severe acute respiratory syndrome coronavirus 2 (SARS-CoV-2) has evolved into a global crisis. We have seen unprecedented rapid development, testing, and emergency use authorization (EUA) of highly effective COVID-19 vaccines, which brought hard-won hope to end the pandemic. However, the success of a vaccine depends not only on its efficacy but also on its acceptance, and a vaccine refusal rate greater than 10% is estimated to significantly impede the attainments of population benefits (1). Therefore, vaccine hesitancy is an important threat to global health, especially during the COVID-19 pandemic (2, 3).

China has achieved a full COVID-19 vaccination rate of nearly 90% (4, 5) and the prevalence of vaccine hesitancy is reported to be only modest among the general Chinese population (6–11). Nevertheless, the scenario among the population with special health concerns is not quite as optimistic. For example, pregnant women have a much lower willingness and more concerns about vaccination (12–16), although they have an increased risk of both maternal and fetal COVID-19 related morbidity and mortality (17). Emerging studies have been initiated to evaluate the safety and effectiveness of periconceptional vaccination on maternal and neonatal outcomes (18–20), as well as the effects of the COVID-19 vaccine on human fertility (21–23) and *in vitro* fertilization (IVF) treatment outcomes (24–28). To date, no evidence has shown that COVID-19 vaccination in the periconception or prenatal period is associated with an increased risk of reproductive health. However, misinformation and conspiracy claims linking COVID-19 vaccines to infertility or adverse reproductive outcomes are widespread on social media, leading to vaccine hesitancy among couples of reproductive age, especially those confronted with infertility (29).

According to data from the Seventh National Census, the total fertility rate in China has reached 1.3 in 2020, which indicated that China is facing the risk of falling into the low fertility trap (30). Recent studies showed further decline in fertility intention during the COVID-19 pandemic (31, 32). Meanwhile, infertility affects 10-15% of couples (33). High prevalence of infertility, the end of the one-child policy and social shifts have increased demand for fertility treatments in China (34). So, it is important to learn about the difficulties that infertile couples are facing during the pandemic.

Thus, we conducted a hospital-based cross-sectional survey to investigate the prevalence of vaccine hesitancy and its associated factors among infertile couples. We also investigated the coverage rate of the primary COVID-19 vaccination, and its influencing on fertility treatments.

## Materials and methods

### Participants

We conducted a confidential, voluntary survey between September 22, 2021, and December 1, 2021, at the Center for Reproductive Medicine at Tianjin Central Hospital of Obstetrics and Gynecology.

Participants were recruited from the waiting room of the Center for Reproductive Medicine. All patients in the waiting room were invited. For all patients who voluntarily to participate, face-to-face interviews were conducted by trained nurses and medical interns using a unified questionnaire. The inclusion criteria of participants included: seeking fertility treatment, having no communication difficulties, and consenting to participate in the survey. Pregnant women coming for prenatal care and their partners were excluded. Patients infected by SARS-Cov-2 were also excluded.

The study was conducted in accordance with the principles of the Declaration of Helsinki, and the study protocol was approved by the ethics committee of Tianjin Central Hospital of Obstetrics and Gynecology (Approval Number: ZY2021004). Informed consent was obtained from each participant prior to data collection.

### Survey instrument

The questionnaire was developed based on the theoretical frameworks of vaccination hesitancy and previous studies related to COVID-19 vaccine acceptance in the general population (6, 9, 35). We collected information on individual demographic characteristics, socio-economic characteristics, history of influenza vaccination, perceptions of the COVID-19 pandemic, and vaccines. In response to the specific concerns of infertile couples and reproductive medicine clinicians, we included questions targeting infertility-related medical history and perception of the association between COVID-19 vaccines and preconception care. To assess attitudes toward the primary and booster vaccinations, we designed two questions referring to the Oxford COVID-19 Vaccine Hesitancy Scale (36): The acceptance or hesitancy status was assessed on a scale of 1 to 5, including (1) absolutely willing to, (2) willing to, (3) not sure, (4) unwilling to and (5) totally against. Options (1) and (2) were defined as “Acceptance” while options (3), (4), and (5) were merged into “Hesitancy”. We also collected information on the status of primary vaccination, postponing of the fertility treatment after vaccination or reasons for delaying the primary vaccination, etc.

### Patient consultations

For all patients visiting our center, we provided uniform information about COVID-19 vaccines based on officially issued expert advice (37–40): no evidence related COVID-19 vaccines to adverse reproductive outcomes, and periconceptional vaccination is recommended for those without contradictions. However, infertile couples are advised to start assisted reproduction technology (ART) treatment one month after immunization for prudential reasons. In addition, for patients who experienced adverse events associated with the vaccination, the ART treatment should be suspended.

### Statistical analysis

Descriptive statistics were presented using mean and standard deviation (SD) for continuous variables and proportion for categorical variables. Student’s t-tests or Chi-square tests were carried out to test differences across groups. Univariate logistic models were used to identify potential predictors of COVID-19 vaccine hesitancy and vaccination status. Factors with a *P*-value < 0.05 were included in the multivariate model. Goodness-of-fit of the logistic models were checked by the Hosmer Lemeshow test. The correlation between COVID-19 vaccine hesitancy and the postpone time of fertility treatment was tested by Cochran-Mantel-Haenszel test. Data were analyzed using the SAS 9.4 statistical package (SAS Institute, Inc., Cary, North Carolina, USA). All tests were two-sided, and *P* < 0.05 was considered statistically significant.

## Results

Among the 1000 individuals who were willing to participate in the interview, 10 participants withdrew halfway, and three participants were excluded due to a previous diagnosis of COVID-19. The 10 dropouts had similar age but lower social-economic status compared to those included in the final analysis (**Table S1** in the Supplementary Material).

The overall coverage rate of the primary COVID-19 vaccine was 67.68%, and none of the participants received booster vaccinations by the time of the survey. As shown in **Table 1**, the average age of the study participants was 32.33 ± 4.37 years. The majority of the participants (87.94%) were women. Compared to those who delayed the primary vaccination, participants who had already received the primary vaccination were more likely to have a college degree or higher, to be employed, to have a higher income, and to have a history of influenza vaccination. Patients who did not receive the primary vaccination had a longer duration of infertility, and more of them were undergoing the IVF treatment. Social media (WeChat, Sina Weibo, Tik Tok, etc.) was the leading source of information about the epidemic and the vaccines. However, the proportion of those who received information from their workplaces was higher among those who had been vaccinated, and they showed more trust in their information sources. The proportion of reporting concerns regarding the influence of the COVID-19 vaccine on pregnancy was 48.95% among those vaccinated and 88.40% among those who were unvaccinated (*P*<0.001). In addition, among the 609 participants who concerned about the influence of the vaccine on pregnancy, 243 (39.9%) believed that the influence would come from both parents while the rest 366 (60.1%) thought the influence would only come from the maternal side.

**Table 1:** Characteristics of participants by vaccination status of the primary COVID-19 vaccine.

	All participants	Vaccinated	Delayed	*P-*value
No. of participants, n (%)	987	668 (67.68)	319 (32.32)	
**Demographic characteristics**				
Age, years	32.33 ± 4.37	32.47 ± 4.31	32.05 ± 4.48	0.158
Female, n (%)	868 (87.94)	580 (86.83)	288 (90.28)	0.119
Education				<0.001
Below high school	182 (18.44)	99 (14.82)	83 (26.02)	
High school	154 (15.60)	91 (13.62)	63 (19.75)	
College or above	651 (65.96)	478 (71.56)	173 (54.23)	
Career, n (%)				<0.001
Government/public institution	133 (13.48)	115 (17.22)	18 (5.64)	
Enterprises	437 (44.28)	318 (47.60)	119 (37.30)	
Self-employed/Farmers	232 (23.50)	141 (21.11)	91 (28.53)	
Unemployed	185 (18.74)	94 (14.07)	91 (28.53)	
Annual household income per capita, CNY	57723 ± 40516	60073 ± 40675	52804 ± 39798	0.008
History of influenza vaccine, n (%)	353 (35.76)	267 (39.97)	86 (26.96)	<0.001
**Clinical characteristics of infertility**				
Duration of infertility	3.55 ± 2.44	3.43 ± 2.35	3.79 ± 2.62	0.039
Type of infertility				0.923
Primary	632 (64.16)	428 (64.26)	204 (63.95)	
Secondary	353 (35.84)	238 (35.74)	115 (36.05)	
Factor of infertility				0.546
Female	502 (50.96)	331 (49.70)	171 (53.61)	
Male	91 (9.24)	64 (9.61)	27 (8.46)	
Both	105 (10.66)	69 (10.36)	36 (11.29)	
Unexplained	287 (29.14)	202 (30.33)	85 (26.65)	
Therapy				<0.001
Expectation/Monitoring/NC-IUI	330 (33.43)	251 (37.57)	79 (24.76)	
OS/OS-IUI	92 (9.32)	61 (9.13)	31 (9.72)	
IVF	368 (37.28)	236 (35.33)	132 (41.38)	
FET	197 (19.96)	120 (17.96)	77 (24.14)	
**Attitudes toward the COVID-19 pandemic and the vaccine**				
Main source of information				
Television/radio/newspaper	424 (42.96)	291 (43.56)	133 (41.69)	0.579
Academic reports/papers/communications	47 (4.76)	30 (4.49)	17 (5.33)	0.563
Propaganda from the workplace	194 (19.66)	162 (24.25)	32 (10.03)	<0.001
Social media (WeChat, Sina Weibo, Tik Tok, etc.)	691 (70.01)	448 (67.07)	243 (76.18)	0.004
Confidence in information source				0.002
Very high	135 (13.68)	105 (15.72)	30 (9.40)	
High	703 (71.23)	474 (70.96)	229 (71.79)	
Not sure	138 (13.98)	82 (12.28)	56 (17.55)	
Low	11 (1.11)	7 (1.05)	4 (1.25)	
Very low	0	0	0	
Severity of the pandemic’s influence on your life				0.434
Very high	103 (10.44)	67 (10.03)	36 (11.29)	
High	225 (22.80)	159 (23.80)	66 (20.69)	
Moderate	408 (41.34)	261 (39.07)	147 (46.08)	
Modest	143 (14.49)	105 (15.72)	38 (11.91)	
None	108 (10.94)	76 (11.38)	32 (10.03)	
Fear for influence on pregnancy	609 (61.70)	327 (48.95)	282 (88.40)	<0.001
Hesitancy in the primary vaccination, n (%)	171 (17.33)	64 (9.58)	107 (33.54)	<0.001
Hesitancy in the booster vaccination, n (%)	253 (25.63)	139 (20.81)	114 (35.74)	<0.001

NC, natural cycle; OS, ovarian stimulation; IUI, in uterus insemination; IVF, *in vitro* fertilization; FET, frozen-thawed embryo transfer.

**Figure 1:**
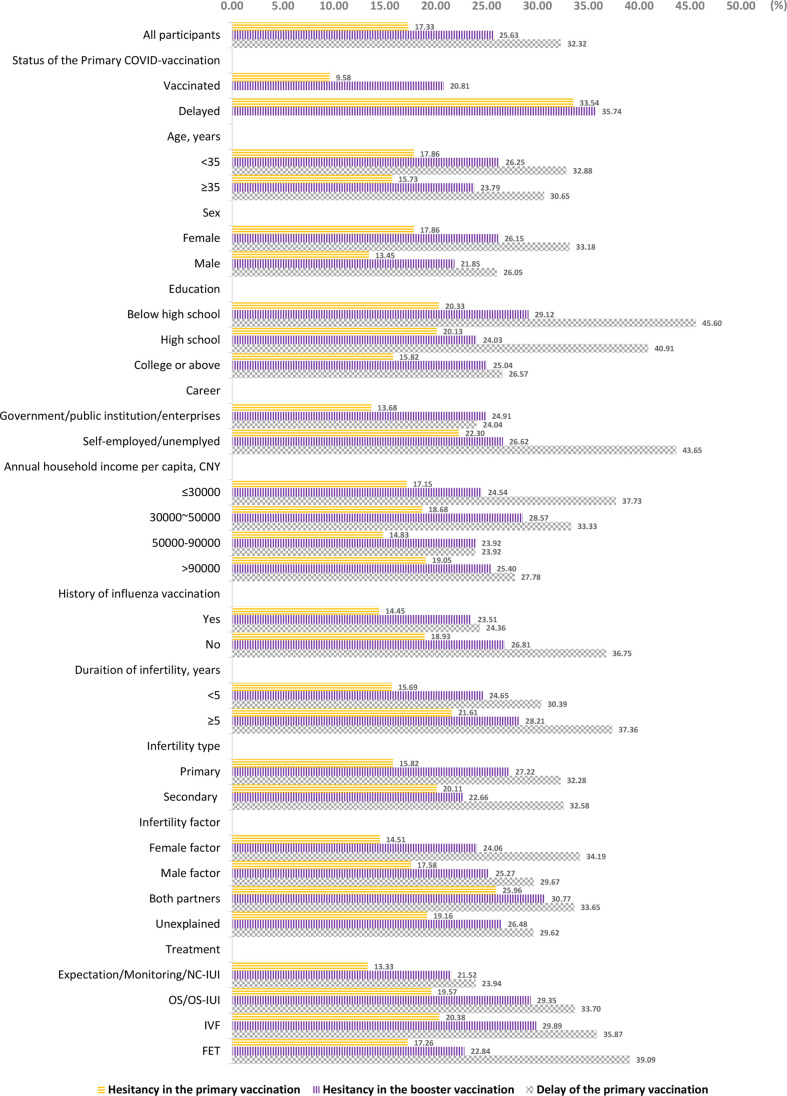
Prevalence of COVID-19 vaccine hesitancy and delay of the primary vaccination.

The prevalence of hesitancy in COVID-19 vaccine and delay in the primary vaccination are presented in **Figure 1**. Overall, 17.33% of all participants reported hesitancy in the primary vaccination, and 25.63% reported hesitancy in the booster vaccination. Furthermore, 9.58% of those who received the vaccination still reported hesitancy regarding the primary vaccination, and the proportion was 20.81% for the booster vaccination. The proportions of reporting hesitancy in primary and booster vaccinations among those who were unvaccinated were 33.54% and 35.74%, respectively. When categorized by demographic and clinical characteristics, the prevalence of hesitancy in the primary vaccination ranged from 13.33% to 25.96% among subgroups with different sociodemographic and clinical characteristics, the prevalence of hesitancy in the booster vaccination ranged from 21.52% to 30.77%, and the delay of the primary vaccination ranged from 23.92% to 45.60%.

According to the univariate analyses (**Table 2**), hesitancy in the primary vaccination was positively associated with the duration of infertility, infertility factor of both couples, ongoing IVF treatment, lower trust in information sources, lower grading of vaccine safety and effectiveness, and belief in the influence of the vaccine on pregnancy. Besides these factors, hesitation in booster vaccination was inversely associated with the immunization status of the primary vaccine but positively associated with reported changes in menstrual cycles among female participants. Delay of the primary vaccination was inversely associated with higher education levels, higher income, and acceptance of influenza vaccine while it was positively associated with a longer duration of infertility, ongoing IVF or FET treatment, lower trust in information sources, lower evaluation of vaccine safety or effectiveness and belief in vaccine influence on pregnancy.

**Table 2:** Associated factors of COVID-19 vaccine hesitancy and coverage rate (univariate model).

	Hesitancy in the primary vaccination		Hesitancy in the booster vaccination		Delay of the primary vaccination	
	OR (95% CI)	*P-*value	OR (95% CI)	*P-*value	OR (95% CI)	*P-*value
**Demographic characteristics**						
Age, per year increase	1.00 (0.96-1.04)	0.985	0.99 (0.96-1.02)	0.492	0.98 (0.95-1.01)	0.206
Female vs male	1.39 (0.80-2.42)	0.247	1.25 (0.79-1.98)	0.347	1.39 (0.90-2.15)	0.134
Education						
Below high school	Reference		Reference		Reference	
High school	0.98 (0.57-1.67)	0.550	0.76 (0.47-1.25)	0.450	0.83 (0.54-1.28)	0.220
College or above	0.73 (0.48-1.11)	0.083	0.80 (0.56-1.16)	0.576	0.43 (0.31-0.61)	<0.001
Annual household income per capita, CNY						
≤30000	Reference		Reference		Reference	
30000~50000	1.11 (0.74-1.66)	0.541	1.23 (0.86-1.74)	0.206	0.83 (0.60-1.14)	0.248
50000~90000	0.84 (0.53-1.34)	0.264	0.94 (0.64-1.40)	0.457	0.52 (0.36-0.76)	<0.001
>90000	1.13 (0.67-1.90)	0.547	1.04 (0.66-1.66)	0.977	0.64 (0.41-0.99)	0.044
Employed, Y vs N	0.57 (0.39-0.85)	0.006	1.01 (0.71-1.43)	0.975	0.49 (0.35-0.68)	<0.001
Influenza vaccination, Y vs N	0.72 (0.48-1.08)	0.110	0.84 (0.60-1.18)	0.314	0.59 (0.43-0.82)	0.002
**Clinical characteristics of infertility**						
Duration of infertility, ≥5 vs <5 years	1.65 (1.10-2.46)	0.015	1.38 (0.97-1.96)	0.071	1.75 (1.25-2.44)	0.001
Type of infertility, secondary vs primary	1.34 (0.96-1.88)	0.089	0.78 (0.58-1.06)	0.117	1.02 (0.77-1.35)	0.872
Factor of infertility						
Female	Reference		Reference		Reference	
Male	1.27 (0.70-2.31)	0.651	1.08 (0.64-1.80)	0.705	0.81 (0.50-1.32)	0.605
Both	2.17 (1.32-3.58)	0.020	1.46 (0.92-2.31)	0.185	0.97 (0.62-1.52)	0.602
Unexplained	1.42 (0.96-2.08)	0.965	1.15 (0.82-1.60)	0.937	0.80 (0.58-1.09)	0.386
Therapy						
Expectation/Monitoring/NC-IUI	Reference		Reference		Reference	
OS/OS-IUI	1.58 (0.86-2.89)	0.138	1.52 (0.90-2.55)	0.117	1.61 (0.98-2.66)	0.061
IVF	1.66 (1.11-2.50)	0.014	1.56 (1.10-2.20)	0.012	1.78 (1.28-2.47)	<0.001
FET	1.36 (0.83-2.21)	0.221	1.08 (0.71-1.65)	0.722	2.04 (1.39-2.99)	<0.001
**Awareness and knowledge of the COVID-19 pandemic and the vaccine**						
Confidence in information source	1.73 (1.25-2.41)	0.001	1.54 (1.15-2.05)	<0.001	1.32 (1.00-1.73)	0.049
Severity of the pandemic’s influence on life	0.89 (0.76-1.03)	0.122	3.34 (2.51-4.43)	0.159	1.05 (0.93-1.18)	0.446
Evaluation of vaccine safety	6.50 (4.51-9.38)	<0.001	2.25 (1.82-2.79)	<0.001	1.53 (1.21-1.93)	<0.001
Evaluation of vaccine effectiveness	2.84 (2.21-3.64)	<0.001	2.95 (2.05-4.24)	<0.001	1.16 (0.96-0.140)	0.125
Influence on pregnancy	3.21 (2.04-5.03)	<0.001	2.81 (2.03-3.90)	<0.001	7.21 (4.77-10.89)	<0.001
Hesitancy in the primary vaccination	NA	NA	NA	NA	4.70 (3.17-6.97)	<0.001
Hesitancy in the booster vaccination	NA	NA	NA	NA	2.08 (1.48-2.92)	<0.001
**Characteristics of primary vaccination**						
Vaccination	NA	NA	0.48 (0.34-0.67)	<0.001	NA	NA
Side effects	NA	NA	1.24 (0.77-2.00)	0.378	NA	NA
Change in menstrual cycle characteristics ^‡^	NA	NA	2.40 (1.10-5.22)	0.028	NA	NA

^‡^, Among women only. NC, natural cycle; OS, ovarian stimulation; IUI, in uterus insemination; IVF, *in vitro* fertilization; FET, frozen-thawed embryo transfer; OR, odds ratio; CI, confidence interval; NA, not applicable.

Multivariate models were constructed using variables with *P*-values of <0.05 in the univariate analysis (**Table 3**). Hesitancy in the primary vaccination was associated with unexplained infertility (OR: 1.77, 95% CI: 1.05-2.98), undergoing IVF treatment cycles (OR: 2.17, 95% CI: 1.22-3.89), worrisome about vaccine safety (OR: 4.13, 95% CI: 2.66-6.42), effectiveness (OR: 1.62, 95% CI: 1.15-2.28) and influence on pregnancy (OR: 2.80, 95% CI: 1.68-4.67). Similarly, hesitancy in the booster vaccination was associated with undergoing IVF treatment cycles (OR: 1.49, 95% CI: 1.02-2.17), worrisome about vaccine safety (OR: 2.05, 95% CI: 1.52-2.78), effectiveness (OR: 1.56, 95% CI: 1.23-1.98) and influence on pregnancy (OR: 2.16, 95% CI: 1.49-3.13). Delay of the primary vaccination was positively associated with influence on pregnancy (OR: 7.78, 95% CI: 5.01-12.07) and negatively associated with having a degree of college or above (OR: 0.49, 95% CI: 0.27-0.87), and a history of influenza vaccination (OR: 0.67, 95% CI: 0.46-0.98). *P* values of the Hosmer Lemeshow test for all three models were larger than 0.05, which indicated that the models were good fits.

**Table 3:** Associated factors of COVID-19 vaccine hesitancy and coverage rate (multivariate model).

	OR (95% CI)	*P-*value
**Hesitancy in the primary vaccination**		
Employed vs unemployed	1.43 (0.89-2.30)	0.140
Duration of infertility, ≥5 vs <5 years	1.09 (0.67-1.79)	0.723
Factor of infertility		
Female	Reference	
Male	1.06 (0.49-2.30)	0.876
Both	1.73 (0.87-3.44)	0.118
Unexplained	1.77 (1.05-2.98)	0.032
Therapy		
Expectation/Monitoring/NC-IUI	Reference	
OS/OS-IUI	1.44 (0.61-3.39)	0.408
IVF	2.17 (1.22-3.89)	0.009
FET	1.32 (0.66-2.61)	0.431
Confidence in information source	1.04 (0.70-1.55)	0.838
Evaluation of vaccine safety	4.13 (2.66-6.42)	<0.001
Evaluation of vaccine effectiveness	1.62 (1.15-2.28)	0.006
Influence on pregnancy	2.80 (1.68-4.67)	<0.001
Goodness-of-fit	χ2 = 8.9796	0.344
**Hesitancy in the booster vaccination**		
Therapy		
Expectation/Monitoring/NC-IUI	Reference	
OS/OS-IUI	1.25 (0.70-2.22)	0.448
IVF	1.49 (1.02-2.17)	0.038
FET	0.85 (0.54-1.35)	0.489
Confidence in information source	1.15 (0.87-1.53)	0.328
Evaluation of vaccine safety	2.05 (1.52-2.78)	<0.001
Evaluation of vaccine effectiveness	1.56 (1.23-1.98)	<0.001
Influence on pregnancy	2.16 (1.49-3.13)	<0.001
Primary vaccination	0.74 (0.52-1.05)	0.089
Goodness-of-fit	χ2 = 4.3909	0.820
**Delay in primary vaccination**		
Education		
Below high school	Reference	
High school	0.80 (0.44-1.45)	0.456
College or above	0.49 (0.27-0.87)	0.016
Annual household income per capita, CNY		
≤30000		
30000~50000	1.04 (0.67-1.62)	0.877
50000-90000	0.90 (0.54-1.50)	0.685
>90000	1.20 (0.68-2.14)	0.685
Employed, Y vs N	1.48 (0.94-2.35)	0.090
Influenza vaccination, Y vs N	0.67 (0.46-0.98)	0.037
Duration of infertility, ≥5 vs <5 years	0.74 (0.50-1.10)	0.140
Therapy		
Expectation/Monitoring/NC-IUI	Reference	
OS/OS-IUI	1.37 (0.71-2.64)	0.341
IVF	1.49 (0.96-2.30)	0.075
FET	1.52 (0.92-2.50)	0.104
Confidence in information source	1.11 (0.81-1.53)	0.520
Evaluation of vaccine safety	1.15 (0.88-1.51)	0.315
Influence on pregnancy	7.78 (5.01-12.07)	<0.001
Goodness-of-fit	χ2 = 8.8943	0.351

NC, natural cycle; OS, ovarian stimulation; IUI, in uterus insemination; IVF, *in vitro* fertilization; FET, frozen-thawed embryo transfer; OR, odds ratio; CI, confidence interval.

Among the 668 participants who received the primary vaccination, 111 (11.25%) reported side effects including redness, swelling and soreness at the injection site, fatigue, headache, nausea or inappetence, and fever. Additionally, 29 (5%) women reported changes in their menstrual cycle characteristics after vaccination. A total of 108 (16.17%) participants reported that they postponed their fertility treatment for more than two months after vaccination (**Table 4**). The majority (95.30%) of those who did not receive the primary vaccination declared that they delayed vaccination due to their preconception plan. Both hesitancy in the primary and the booster vaccination correlated with prolonged postpone of the fertility treatment (*P = 0.020* and <*0.001*, respectively).

**Table 4:** Information on the primary vaccination.

	Vaccinated participants
Primary vaccination	668
Completeness of vaccination	
Complete	192 (28.74)
Not complete	476 (71.26)
Type of vaccines	
CNBG	261 (39.07)
Sinovac	331 (49.55)
Adenovirus vector	53 (7.93)
CHO	23 (3.44)
Side effects	
None	557 (83.38)
Yes	111 (16.62)
Type of side effects	
Redness, swelling or soreness at the injection site	71 (7.19)
Fatigue	41 (6.14)
Headache	27 (4.04)
Nausea or inappetence	10 (1.50)
Fever	25 (3.73)
Change in menstrual cycle characteristics^‡^	29 (5.00)
Postpone of ART treatment after vaccination	
≤1 month	560 (83.53)
2-3 month	68 (10.18)
4-6 months	21 (3.14)
>6 months	19 (2.84)

^‡^Among women only.

## Discussion

To the best of our knowledge, this was the first study to investigate the COVID-19 vaccine coverage rate, prevalence of hesitancy, and associated factors among infertile couples seeking fertility treatment. According to our survey, infertile couples had a higher level of hesitancy in the COVID-19 vaccine and a lower vaccine coverage rate compared to the general population. The hesitancy rates and coverage rates varied between gender and other socio-economic groups. Clinical characteristics of infertility, ongoing IVF treatment, concerns regarding vaccine safety and effectiveness, and fear for influence on pregnancy were major factors associated with vaccine hesitancy and the delay of the primary vaccination. Covid-19 vaccine hesitancy was also correlated with longer periods of fertility treatment suspension.

This survey showed high prevalence of hesitancy in COVID-19 vaccine and low coverage rate of the primary vaccination among couples seeking fertility treatment. Many epidemiological studies have been carried out on the attitudes toward the COVID-19 vaccines (**Table S2** in the supplementary material) (15, 16, 41–44). According to these studies, the rates of COVID-19 vaccine hesitancy varied between countries and between different time points. Studies showed that the COVID-19 vaccines hesitancy among general Chinese population was modest and decreased over time (6–11). According to a national survey in August 2021 (which was the closest to our survey time), the hesitancy rates for primary/booster vaccination were 8.4% nationwide (6) but was approximately 15% in Tianjin. The pandemic had been well-controlled in Tianjin, which might explain the lower enthusiasm. Even so, the hesitancy rate of infertile patients in Tianjin in our survey was relatively higher than that among the general population, ranging from 13.3% to 26.0% for the primary vaccination and 21.5% to 30.8% for the booster vaccination. At the time of this survey, the vaccination coverage rate had accumulated to over 80% among general population in Tianjin, but only 68% among infertile couples in this survey. The reluctance to receive the vaccine among couples of reproductive ages was consistent with previous findings among pregnant women (13, 15, 16). These findings confirmed that the couples with child bearing plan would be more cautious about taking the vaccines. However, the reluctance of taking vaccines would subsequently increase the risk of adverse maternal and neonatal outcomes.

Consistent with findings among the general population, concerns about the vaccine safety and effectiveness were factors that significantly associated with vaccine hesitancy (43, 45, 46). Infertility-related risk factors for vaccine hesitancy included longer duration of infertility, infertility factor of both partners, ongoing IVF treatment, and concerns about potential influence on pregnancy. Fear for influence on pregnancy was also the predominant reason for delay of the primary vaccination. These factors may be associated with significant depressive and anxiety disorders, which occurred widely among infertile patients. A recent survey found that despite the immense and ubiquitous impact of COVID-19, infertile women still ranked infertility as the greatest stressor (47). These findings indicated that psychological distress caused by infertility can hardly be surpassed by concerns about the pandemic. As fertility treatments are often not successful on the first attempt and may require numerous attempts to achieve an ongoing pregnancy, the delay period of vaccination due to ongoing ART treatment could not be predicted. This would be detrimental to the establishment of a herd immune barrier. Furthermore, considering the reluctance of vaccination during gestation, this could lead to both pregnant women and their fetuses being unprotected.

Vaccine hesitancy also led to a prolonged delay of fertility treatment after the vaccination in this study. Due to the disruptions, prolonged lockdowns, childcare by parents following school closures, deteriorating economic outlooks and uncertainties caused by the pandemic, couples are increasingly deciding to postpone childbearing (48). China is facing declines in births pointing to a baby bust and we have seen the least babies born in 2021 ever since 1949. At our center, daily outpatient visits and treatment cycles have dropped dramatically since the COVID-19 outbreak. This slump continued even after the pandemic has been controlled. According to our survey, fear for the influence of COVID-19 vaccine could contribute to this situation partially. However, for women over mid-thirties or with diminished ovarian reserve, their fertility and the opportunity for treatment success is time sensitive. Consequently, the indefinite suspension of fertility treatment could be devastating, and had a large emotional, psychological, and financial impacts. It is important to make a time-saving schedule and arrange the fertility treatment and the vaccination reasonably. Individualized consultation and share-decision making process may help infertile couples rationalize their child-bearing plan and weather the bad times.

Furthermore, the majority of the study participants were women, and they had higher prevalence of vaccine hesitancy and lower coverage rate of the primary vaccination. This was consistent with previous studies indicated higher vaccine hesitancy among women (6, 43). We noted that 29 (5%) women reported menstrual abnormalities after the primary vaccination, which was also mentioned by several previous studies (49, 50). Although it was unknown whether the menstrual abnormalities were cause by potential biological influence of the vaccine or by stress and psychological distress, it was known that women were going through this uncomfortableness, which might also influence on their child-bearing plan. In addition, we found more participants mistakenly assumed only women getting vaccinated would have adverse influence on pregnancy. Besides, lower level of education, lower income, unemployment status and no experience of vaccination against influenza were associated with delay of the COVID-19 vaccine in our survey. These findings supported theory put forward by earlier studies that the impact of COVID-19 on fertility of different socio-economic status might be different (48). In other words, different socio-economic status might influence the acceptance of the vaccination, and might in turn, influence the child-bearing plan and post-pandemic fertility trajectories. These findings indicated health inequalities related to gender and socio-economic status regarding to COVID-19 vaccines.

The effects of the COVID-19 vaccines have been drawing broad attention among clinicians and researchers. No theoretical or actual adverse effects have been prompted (21–23). No detrimental effects were observed among women who got vaccinated during pregnancy (18–20). Two recent cohorts among infertile couple in China also revealed that vaccination before ovarian stimulation had no effects on IVF outcomes (18–20, 27, 28). Thus, we recommended that clinicians could be more positive and encourage infertile couples with no other contradiction to take the vaccines as early as possible. It is necessary to carry out targeted education program by health professionals to publicize the benefits of periconception vaccination, reduce the misconception and resistance to COVID-19 vaccine among infertile couples, especially among women and patients with low socio-economic status. Individualized consultation and share-decision making process may help infertile couples rationalize their child-bearing plan and weather the bad times.

This survey had several limitations. First, this was a single-center study conducted in a municipality in China, where mainly inactivated viral vector vaccinations were provided. Second, this survey was conducted among couples who were still seeking infertility treatment during the pandemic. Thus, selection bias due to the recruitment of the participants could not be neglected and the generalization of our findings to all infertile couples should be made with cautious.

In conclusion, our survey showed a high prevalence of COVID-19 vaccine hesitancy and delay in vaccination among infertile couples. These findings showed that infertile couples were still confused about whether or when should they get vaccinated and how long should they suspend the fertility treatment after the vaccination. It is necessary to carry out targeted education program by health professionals to publicize the benefits of periconception vaccination, and to reduce the resistance to COVID-19 vaccine among infertile couples.

## Data availability statement

The data that support the findings of this study are available from the corresponding author, upon reasonable request.

## Ethics statement

The studies involving human participants were reviewed and approved by Ethics Committee of Tianjin Central Hospital of Obstetrics and Gynecology. The patients/participants provided their written informed consent to participate in this study.

## Author contributions

XW conceptualized the hypothesis, designed the work, analyzed the data, interpreted the results and drafted the article; HW, AD, JW, JS, YFZ, YSZ, and JM designed the work, and interpreted the results; JM, JL and WM interviewed the participants, and enter the data; HL conceptualized the hypothesis, designed the work, and interpreted the results; all authors revised the article and approved the final version for publication.

## Funding

This work was supported by the National Natural Science Foundation of China (grant number: 82103928) from the Ministry of Science and Technology of China.

## Acknowledgments

We acknowledge all medical and research staff of our team at the Center for Reproductive Medicine, Tianjin Central Hospital of Obstetrics and Gynecology, and a special thanks to all the study participants.

## Conflict of interest

The authors declare that the research was conducted in the absence of any commercial or financial relationships that could be construed as a potential conflict of interest.

## Publisher’s note

All claims expressed in this article are solely those of the authors and do not necessarily represent those of their affiliated organizations, or those of the publisher, the editors and the reviewers. Any product that may be evaluated in this article, or claim that may be made by its manufacturer, is not guaranteed or endorsed by the publisher.
