# Supplementary material for: COVID-19 vaccine hesitancy and associated factors among infertile couples undergoing assisted reproductive treatment
Source: Front Immunol. 2022 Sep 27;13:973600. doi: 10.3389/fimmu.2022.973600 (PMC9552881; doi:10.3389/fimmu.2022.973600)
Supplement: Supplementary file 1 [file DataSheet_1.pdf]

Supplementary Material

Table S1. Comparison of baseline characteristics between respondents and non-respondents.

	**Respondents**	**Non-respondents**
No. of participants, n (%)	987	10
Age, years	32.33 ± 4.37	32.49 ± 3.65
Female, n (%)	868 (87.94)	9 (90.00)
Education		
Below high school	182 (18.44)	8 (80.00)
High school	154 (15.60)	1 (10.00)
College or above	651 (65.96)	1 (10.00)
Career, n (%)		
Government/public institution	133 (13.48)	1 (10.00)
Enterprises	437 (44.28)	0
Self-employed/Farmers	232 (23.50)	1 (10.00)
Unemployed	185 (18.74)	8 (80.00)
Annual household income per capita, CNY	57723 ± 40516	29500 ± 15134

Table S2. A summary of previous systematic reviews of epidemiology studies on COVID-19 vaccine hesitancy.

Study ID	No of studies	Study design	Study Period	Sample size	Region/Country	Population	Attitude estimation	Potential influencing factors
Joshi, A., et al. (2021). Front Public Health 9: 698111.	22	CS	Update to 2020.12.15	316 to 13426	27 different countries	General population (n=17); HCW (n=3); parents (n=2).	Acceptance: rate*: 54% to 86%	Gender, age, education, and occupation, trust in authorities, risk perception of COVID-19 infection, vaccine efficacy, current or previous influenza vaccination, and vaccine safety
Salomoni, M. G., et al. (2021). Vaccines (Basel) 9(8).	100	CS (n=86), LS (n=8), and 6 did not report.	2019.11-2021.3	87 to 28629	Africa (n=3), America (n=35), Asia (n=24), Australia (n=4), Europe (n=31), international context (n=7).	General population (n=49), HCW (n=22), patients with chronic diseases (n=9), pregnant women (n = 1), others (19) included students, industry workers, etc.	Acceptance rate: 4.2% to 94.3%	Overall, this study demonstrated significant differences in terms of VH in the general population and in the specific subgroups examined according to geographical, demographic factors, as well as associated comorbidities.

Study ID	No of studies	Study design	Study Period	Sample size	Region/Country	Population	Attitude estimation	Potential influencing factors
Cascini, F., et al. (2021). EClinical Medicine 40: 101113.	209	CS	Update to 2021.7.5	103 to 36220	Africa (n=17), America (n=48), Asia (n=78), Australia (n=5), Europe (n=53), and international context (n=8).	General population.	Acceptance rate: 27.7% to 94.3%	Vaccine acceptance rates ranged considerably between countries and between different time points. Factors associated with increased hesitancy included: having negative perception of vaccine efficacy, safety, convenience, and price; women, younger participants, and people who were less educated, had lower income, had no insurance, living in a rural area, and self-identified as a racial/ethnic minority.
Shakeel, C. S., et al. (2022). Vaccines (Basel) 10(1).	81	CS	Update to 2021.7.31	248 to 13426	50 different countries	General population (n=57), HCW (n=16), others (n=8) included adolescents, parents, pregnant women, T2DM patients, etc.	Acceptance rate: 21.4%-93.3%	Low vaccine acceptance was associated with low levels of education and awareness, and inefficient government efforts and initiatives. Furthermore, poor influenza-vaccination history, as well as conspiracy theories relating to infertility and misinformation about the COVID-19 vaccine on social media also resulted in vaccine hesitancy.

Study ID	No of studies	Study design	Study Period	Sample size	Region/Country	Population	Attitude estimation	Potential influencing factors
Bhattacharya, O., et al. (2022). BMJ Open 12(8): e061477.	17	CS	2020.12-2022.4	25147 totally	Asia (n=6), Africa (n=2), North America (n=2) and Europe/Oceania (n=6)	Pregnant women	Acceptance rate: 30% to 77%	High-income countries, participants with fewer than 12 years of education and multiparous women had lower COVID-19 vaccine acceptance.
Rawal, S., et al. (2022). Am J Obstet Gynecol MFM 4(4): 100616.	11	CS or LS	2020.1.1-2022.2.6	93 to 135968	United states	Pregnant women	Acceptance rate: 3% to 65%	Influenza vaccination, communication with a medical professional, age, race, ethnicity, education level, concerns for safety and effectiveness of COVID-19 vaccination, fears of birth defects, unknown long-term health effects on children, and risk of pregnancy loss.

CS: Cross-sectional study; LS: longitudinal study; HCW: health care workers. "*Acceptance rate" was calculated considering both positive and uncertain answers showing willingness to be vaccinated (e.g., "probably yes", "somewhat likely", or "somewhat agree"). Other indicators included confidence rate (same with acceptance rate) and hesitancy rate (opposite to acceptance rate). Slightly heterogeneity existed in the definitions of the original studies.
